# Law Regulating the Practice of Medicine and Surgery in Pennsylvania

**Published:** 1869-05

**Authors:** 


### Law Regulating the Practice of Medicine and Surgery in Pennsylvania.

The following bill was passed by the last Legislature, has been signed by the Governor, and is now a law:

§ 1. That from and after the first day of June, 1869, it shall be unlawful for any person to commence or continue the practice of medicine or surgery in the counties of Erie, York, Lancaster, Crawford, Venango, Warren, Adams, Bucks, Northampton and Lehigh, who has not graduated with the degree of Doctor of Medicine, and received a diploma from a chartered medical college or other institution, authorized to grant diplomas. Provided, that the provisions of this section shall not apply to persons who have been eight years in continuous regular practice, though they may not have graduated as aforesaid.

§ 2. Any person who shall practice, or attempt to practice, medicine or surgery, or shall prescribe for any sick person, or perform any surgical operation for fee or reward in violation of section one of this act, shall be deemed guilty of a misdemeanor, and upon conviction in any court of competent jurisdiction, shall be fined in any sum not less than one hundred dollars, nor more than five hundred dollars, at the discretion of the court, one-half of said fine to be for the use of the informer, and half for the county in which such fines shall be imposed.

§ 3. Any person who shall attempt to practice medicine or surgery, by opening a transient office in any of the aforesaid counties, or who shall, by handbill or other form of written or printed advertisement, assign such transient office or other place to meet persons seeking medical or surgical advice, or prescription, shall before being allowed to practice, as aforesaid, appear before the clerk of the courts of the proper county, and furnish satisfactory evidence to such clerk of the courts that the provisions of section one of this act have been complied with, and shall in addition, take out a license for one year by payment of a license fee, for the use of the proper county, of two hundred dollars: Provided, that the provisions of this act shall not apply to druggists or dentists. And provided further, that physicians commencing practice in any of the aforesaid counties, with the intention of residing permanently therein, shall not be subject to the provisions of section three of this act.

